# Experimental *Trypanosoma cruzi* Infection Induces Pain in Mice Dependent on Early Spinal Cord Glial Cells and NFκB Activation and Cytokine Production

**DOI:** 10.3389/fimmu.2020.539086

**Published:** 2021-01-26

**Authors:** Sergio M. Borghi, Victor Fattori, Thacyana T. Carvalho, Vera L. H. Tatakihara, Tiago H. Zaninelli, Felipe A. Pinho-Ribeiro, Camila R. Ferraz, Larissa Staurengo-Ferrari, Rubia Casagrande, Wander R. Pavanelli, Fernando Q. Cunha, Thiago M. Cunha, Phileno Pinge-Filho, Waldiceu A. Verri

**Affiliations:** ^1^ Department of Pathology, Center of Biological Science, State University of Londrina, Londrina, Brazil; ^2^ Center for Research in Health Science, University of Northern Paraná—Unopar, Londrina, Brazil; ^3^ Departament of Pharmaceutical Sciences, Health Sciences Center, University Hospital, Londrina State University, Londrina, Brazil; ^4^ Department of Pharmacology, Ribeirão Preto Medical School, University of São Paulo, Ribeirão Preto, Brazil

**Keywords:** *Trypanosoma cruzi*, pain, glial cells, NFκB, cytokine

## Abstract

The neglected tropical infirmity Chagas disease (CD) presents high mortality. Its etiological agent *T. cruzi* is transmitted by infected hematophagous insects. Symptoms of the acute phase of the infection include fever, fatigue, body aches, and headache, making diagnosis difficult as they are present in other illnesses as well. Thus, in endemic areas, individuals with undetermined pain may be considered for CD. Although pain is a characteristic symptom of CD, its cellular and molecular mechanisms are unknown except for demonstration of a role for peripheral TNF-α in CD pain. In this study, we evaluate the role of spinal cord glial cells in experimental *T. cruzi* infection in the context of pain using C57BL/6 mice. Pain, parasitemia, survival, and glial and neuronal function as well as NFκB activation and cytokine/chemokine production were assessed. *T. cruzi* infection induced chronic mechanical and thermal hyperalgesia. Systemic TNF-α and IL-1β peaked 14 days postinfection (p.i.). Infected mice presented increased spinal gliosis and NFκB activation compared to uninfected mice at 7 days p.i. Glial and NFκB inhibitors limited *T. cruzi*–induced pain. Nuclear phosphorylated NFκB was detected surrounded by glia markers, and glial inhibitors reduced its detection. *T. cruzi*–induced spinal cord production of cytokines/chemokines was also diminished by glial inhibitors. Dorsal root ganglia (DRG) neurons presented increased activity in infected mice, and the production of inflammatory mediators was counteracted by glial/NFκB inhibitors. The present study unveils the contribution of DRG and spinal cord cellular and molecular events leading to pain in *T. cruzi* infection, contributing to a better understanding of CD pathology.

## Introduction

According to the World Health Organization, more than 1.5 billion people worldwide are infected with neglected tropical diseases. Among them, 8 million people have Chagas disease (CD; American trypanosomiasis) with a mortality rate reaching 10,000 deaths per year (1). More than 25 million people are at risk of infection in endemic areas, and globalization contributes to the propagation of CD to other areas besides America (1). There are two well-established phases of CD upon infection, beginning with an acute phase that starts 6–10 days after infection with the parasite *Trypanosoma cruzi (T. cruzi*) and lasts for about 4–8 weeks, depending on mouse strain and parasite load and strain (2). In the present experimental condition, the parasitemia ends by the 28th day postinfection (p.i.) as demonstrated by previous studies by our group (3). During the acute phase of CD, high numbers of parasites are found in the bloodstream and tissues, and after this period, the parasite and the host reach an immunological balance and a chronic phase settles in. In the chronic phase of CD, parasitemia is substantially reduced, and patients become asymptomatic until major organ pathology develops, affecting, for instance, the heart (1, 2).

Immunoregulatory events found in the acute phase of CD include an intense inflammatory response with high production of inflammatory cytokines and nitric oxide (NO), which are important to orchestrate immune cells to eliminate the parasite (1, 4). These are key mediators in controlling infection because, for instance, tumor necrosis factor-α deficient (TNF-α)^-/-^ and inducible nitric oxide synthase deficient (iNOS)^-/-^ mice are highly susceptible to *T. cruzi* infection (1, 5).

Clinical and epidemiological studies of patients with CD in the acute phase report pain and inflammation as major symptoms (6–11). In studies conducted in endemic areas of Brazil, fever (98%), headache (92.3%), myalgia (80%), swelling of the lower limbs (57.9%), facial edema (57.5%), abdominal pain (54.1%), and painful lumps (17%) were the most reported symptoms (7, 8). However, the cellular and molecular mechanism underlying the pain in CD remains unknown. There is evidence of high levels of peripheral TNF-α in CD (12). TNF-α has a well-established function in triggering hyperalgesia (13). Interleukin (IL)-1β is another hyperalgesic cytokine (14) that is speedily produced in response to *T. cruzi* infection (12, 15). Thus, the systemic elevations in TNF-α and IL-1β production during *T. cruzi* infection may account for the nociceptor neuron sensitization/activation and consequent prominent pain observed in CD patients. Further reinforcing this concept, targeting TNF-α in experimental CD reduces mechanical allodynia during the first week after infection (16). C-X_3_-C motif chemokine ligand 1 (CX_3_CL1) is another important molecule related to neuroinflammation and the central sensitization processes leading to pain, which is crucial for the neuron–glia interface during nervous system pathology (17). Its serum concentration is increased in the acute phase of CD (18); however, whether its signaling *via* C-X_3_-C motif chemokine receptor 1 (CX_3_CR1) in the central nervous system (CNS) has a role in CD pathology is unknown.

In addition to high serum levels of hyperalgesic cytokines, chronic phase CD patients may present severe neurological manifestations, resulting in elevated morbidity and mortality rates (19). Amastigote forms and *T. cruzi* granular antigens (this last one restricted to inflamed foci) can be abundantly detected in CNS (cortex and basal nuclei) in the chronic phase of CD in inbred mice, suggesting potential local actions (20, 21). There seems to occur an active interaction between *T. cruzi* and the immune/nervous system because the *T. cruzi* parasite can be found infecting mouse peripheral glial cells (22–24); brain astrocytes (25–27); and spinal cord astrocytes, microglia, and macrophages (24, 28) as well as human peripheral glial cells (29), astrocytes (25, 30), and neurons (29). In immunocompromised humans, such as patients with human immunodeficiency virus (HIV) and cancer, *T. cruzi* infection results in brain and spinal cord lesions, neurological abnormalities, and parasite detection in the brain and cerebrospinal fluid (25, 31). During the chronic phase of CD, CNS parasitism may rise by reentry of blood parasites or migration of infected macrophages (25). Spinal cord neuropathology occurs with the presence of *T. cruzi*–enhanced disease in mice lacking the IL-12p40 gene compared to wild type (28). Cultured dorsal root ganglia (DRG) and spinal cord neurons of mice infected with *T. cruzi* show altered morphology and can evolve to death that occurs probably by phagocyte overt inflammation in response to parasitism (24, 27). However, the neuro-immune interactions in the spinal cord leading to pain during *T. cruzi* infection are still unaddressed. This lack of information impairs the comprehension of central immune inflammatory events in CD considering that the spinal cord plays a pivotal role in the integrative mechanisms of pain processing.

Our group recently demonstrated the role of spinal cord astrocytes and microglia in neuroinflammation and central processing of pain in another neglected tropical disease, leishmaniasis, contributing to advance in the understanding of neuroimmune interactions that occur in the host in response to the parasite (32). However, although the evidence described above strongly suggests that systemic immune and inflammatory profiles of CD may account for the pain-related symptoms observed in human patients, the central pathophysiological mechanisms of the nociceptive activity in *T. cruzi* infection remains poorly explored. Therefore, the present study investigates the participation of spinal cord glial cells (microglia and astrocytes) in the pathology of experimental CD-induced pain using C57BL/6 mice.

## Methods

### Animals

The experiments were performed on male C57BL/6 mice, 7–13 weeks, resistance prototype to *T. cruzi* infection, weighing between 20 and 30 g from TECPAR (Paraná Technology Institute), PR, Brazil. The choice of exclusively male mice for the study is based on previous findings indicating gender dimorphism in nociception and no effect of glial inhibitors in females in this specie (32). Pathogen-free mice were housed in standard clear plastic cages appended to a ventilated rack (Alesco Indústria e Comércio LTDA, Monte Mor, Sao Paulo, Brazil) with free access to water and food, a light/dark cycle of 12/12 h, and controlled temperature. Shaving and feed provision for the animals was always controlled by a sterilization process. The mice were separated into a maximum of five per cage and were maintained in the vivarium of the Department of Pathology at the State University of Londrina for at least 2 days before the experiments. Mice were used only once and were acclimatized to the testing room at least 1 hour before the experiments, which was conducted during the light cycle. At the end of the experiments, mice were anesthetized with isoflurane 5% only once by inhalation overdose, and terminally euthanized by cervical dislocation followed by decapitation. Animals were monitored regarding welfare-related assessment before, during, and after the experiments. Clinical signs of severe illness, including changes in body weight, erection of the back hairs (reflecting irritation or agitation), diarrhea, lethargy, rapid/shallow breathing, and paralysis, were also properly recorded. Animals presenting clinical signs of severe disease before the end of the model were immediately euthanized using isoflurane 5% followed by cervical dislocation as per the guidelines of the Ethics Committee on Animal Use (CEUA) of the State University of Londrina. The euthanasia process used in the study for sample collection also followed the protocol of anesthesia of animals with isoflurane 5% followed by cervical dislocation according to the CEUA guidelines. Efforts were maintained to narrowly minimize the total number of animals used and their discomfort or suffering.

### Drugs

Isoflurane was obtained from Abbot Park (Lake Bluff, IL, USA). L-2-aminoadipic acid (α-aminoadipate; A7275), minocycline hydrochloride (M9511), ammonium pyrrolidine dythiocarbamate (PDTC; P8765), dimethyl sulfoxide (DMSO; D8418), and tween 80 (P5188) were purchased from Sigma-Aldrich (St. Louis, MO, USA). Mouse neutralizing antibody anti-CX_3_CL1 (AF472) was obtained from R&D System (Minneapolis, MN, USA). Etanercept was obtained from Wyeth (Enbrel^®^, São Paulo, SP, Brazil). IL-1 receptor antagonist was obtained from the National Institute for Biological Standards and Control (IL-1ra; NIBSC, South Mims, Hertfordshire, UK). Vehicle was obtained from LBS Laborasa (sodium chloride 0.9%; São Paulo, SP, Brazil). α-aminoadipate, minocycline, PDTC, etanercept, IL-1ra, antibody anti-CX_3_CL1, and vehicle were injected only once by the intrathecal route (L_4_-L_6_ segment) in a final volume of 5 μL to obtain a local effect. We avoid repeated intrathecal injections because they cause increased nociceptive response *via* prostanoid production (33) and could, thus, interfere with the analysis of the present model. Doses of treatments used in the present model were chosen because they have analgesic effects as demonstrated previously (32). Intrathecal injections were performed in animals under a brief period of anesthesia induced by inhalation of isoflurane 5%.

### *T. cruzi* Infection Protocol, Blood Parasite Count, and Survival Rate Analysis

Protozoan parasite *T. cruzi* (Kinetoplastea; Trypanosomatida; Trypanosomatidae; *Trypanosoma*) infection was induced using Y strain (*TcII*) and was maintained by weekly intraperitoneal inoculation in Swiss mice with 2x10^5^ blood trypomastigotes as described previously (34). Briefly, the blood of animals infected anteriorly was obtained by cardiac puncture under anesthesia without the use of anticoagulants. Samples were then centrifuged followed by standing in 37°C, and serum supernatant containing most parasites was centrifuged again for the resuspension of the pellet using appropriate culture and compounds (GIBCO, Grand Island, NY, USA). Trypomastigotes were derived from the supernatant of *T. cruzi*–infected LLC-Mk2 culture cells (ATCC CCL-7; American Type Culture Collection, Rockville, MD, USA) and grown in appropriate culture (GIBCO, Grand Island, NY, USA). Subconfluent cultures of LLC-Mk2 were infected with 5x10^6^ trypomastigotes. Free parasites were removed after 24 h, and cultures were maintained in medium. Five days after infection, free trypomastigote forms could be found in the cell supernatant, and the infection of animals was performed between the 7th and 8th days afterward by the intraperitoneal route. Blood parasitemia was assessed under standardized conditions by direct microscopic observation at 400x magnification of 50 fields. Counting the number of circulating parasites was performed in a volume of 5 μL of blood collected from the tail vein of infected animals. This result was expressed as the number of parasites per mL^-1^ in the blood samples. Blood parasitemia was evaluated every 7 days, starting on the 7th day of infection, and survival rates were monitored daily. Infection outcomes in mice were monitored daily to assess their health conditions during the experimental period.

### General Experimental Procedures

Mice were infected with *T. cruzi* (5x10^3^ infective trypomastigotes) by one intraperitoneal inoculation, and the following parameters at the respective time points evaluated in comparison to uninfected mice are described in sequence. Mechanical hyperalgesia and thermal hyperalgesia were assessed every 2 days for a period of 28 days. Survival of infected animals was recorded daily, and survival rate plotted every 2 days as a percentage. Blood parasite load was monitored every 7 days (7, 14, 21, and 28 days). Blood of the animals was also used to determine TNF-α and IL-1β protein levels on the same days described for the assessment of parasitemia. Temporal profile (7–28 days p.i.) of glial fibrillary acidic protein (*Gfap*) and ionized calcium-binding adapter molecule 1 (*Iba1*) mRNA expression or protein levels/staining (Western blot and immunofluorescence) were evaluated in spinal cord samples of uninfected and infected mice. The 7th day was selected because, at this time point, we could evaluate the participation of astrocytes (GFAP) and microglia (Iba-1) at the same time using all three approaches if necessary as per the results section. Next, infected animals were treated with vehicle, glial (α-aminoadipate and minocycline), and NFκB (PDTC) inhibitors by the intrathecal route for the evaluation of *Gfap* and *Iba1* mRNA expression, blood parasitemia, and mechanical and thermal hyperalgesia (1–7 h after the treatments) on day 7 p.i.; survival percentage was also assessed during this period to ensure that no animals died during the spinal treatment protocol, so certifying intrathecal treatments would not be harmful to the animals. We opted to evaluate drug treatment effects during 1–7 h because we have previously standardized this approach to study the acute effect of treatments in ongoing hyperalgesia (32). Posteriorly, the effects of intrathecal treatments described above (most effective dose of each inhibitor) upon NFκB activation (7 h after the treatments) as well as double immunofluorescence analysis using antiglial and -p-NFκB antibodies 7 days p.i. were performed. The evaluation of the temporal profile of *Cx3cr1*, *Tnfα*, and *Il1β* mRNA expression in the spinal cord were conducted 7–28 days after the infection. In another round of experiments, mice were treated by the intrathecal route with vehicle, antibody anti-CX_3_CL1, etanercept, and IL-1ra and mechanical and thermal hyperalgesia (1–7 h after the treatments), and parasitemia was investigated at day 7 after the infection; again, survival percentage was assessed during this period to ensure that no animals died during the spinal treatment protocol and that the intrathecal treatments would not be harmful to the animals. After this measurement, infected animals were treated with vehicle, glial (α-aminoadipate and minocycline), and NFκB (PDTC) inhibitors (most effective doses) by the intrathecal route to be evaluated; this time, the *Cx3cr1*, *Tnfα*, and *Il1β* mRNA expression in the spinal cord samples at the 7th day p.i. 7 h after the treatments. The activation of DRG neurons in response to infection alone and co-stimulus with capsaicin were examined using the calcium influx test through confocal microscopy as well as mRNA expression of *Trpv1* (transient receptor potential cation channel subfamily V member 1) at the 7th day p.i. The effects of intrathecal treatments with vehicle, glial (α-aminoadipate and minocycline), and NFκB (PDTC) inhibitors (most effective doses) were also evaluated over *Cx3cl1*, *Cx3cr1*, *Gfap*, *Tnfα*, *Il1β*, and *Cox2* mRNA expression in DRG cells at the day 7 after infection 7 h after the treatments.

### Evaluation of Mechanical Hyperalgesia

Mechanical hyperalgesia was tested in mice as previously described (35). Briefly, in a quiet room, mice were placed in acrylic cages (12 X 10 X 17 cm) with wire grid floors, for at least 30 min before the test for adequate environmental adaptation. Stimulations were performed only when the animals were quiet without exploratory movements or defecation and not resting on their paws. The test consisted of evoking a hind paw flexion reflex with a hand-held force transducer (electronic von Frey esthesiometer; Insight, Ribeirão Preto, SP, Brazil), adapted with a 4.15-mm^2^ (referred to as large probe) contact area polypropylene tip (35). The experimenter applies the probe perpendicularly to the central area of the hind paw with a gradual increase of pressure. The applied pressure to the hind paw surface induces movement of the ankle and knee joints, evoking stretching and/or shortening of leg muscles, promoting a muscle contraction response, which is enough to trigger muscle nociceptive activity (movement-induced hyperalgesia) when the latter is sensitized. The end point was characterized by the removal of the hindlimb followed by clear flinch movements. The measurements were standardized to be always measured on the right paw of the mice. The electronic pressure meter apparatus automatically recorded the intensity of the pressure applied when the paw was withdrawn. The value for the response was an average of three measurements. Only one experimenter started and ended the same test in order to avoid differences in the application of the pressure transducer force. The experimenter was always blinded to the experimental groups. The results are expressed by mechanical threshold in grams (g).

### Evaluation of Thermal Hyperalgesia

The mensuration of thermal hyperalgesia in mice was conducted as described previously (32). Mice were positioned on the heated surface of a hot plate apparatus isolated by containment of a transparent acrylic material (EFF 361, Insight, Ribeirão Preto, SP, Brazil). The temperature on the surface was always maintained at 55 ± 1°C. The reaction time was registered using a conventional chronometer when the mouse presented the behaviors of licking or flinching one of the hind paws. A time limit of 15 s of maximum latency was defined as a cutoff with the intention of avoiding potential tissue injuries. The assessment of thermal hyperalgesia was performed before and after the infection process. The experimenter was always blinded to the experimental groups. The results were expressed by thermal threshold in seconds (s).

### Assessment of Cytokine Levels

The blood of infected mice was collected in sterile microtubes containing the anticoagulant ethylenediamine tetraacetic acid (EDTA) by cardiac puncture following anesthetization with isoflurane 5%. Samples were then centrifuged for separation of plasma, and the resultant supernatant was separated for the tests. TNF-α and IL-1β levels were determined through an enzyme-linked immunosorbent assay (ELISA) test using paired antibodies following the manufacturer’s instructions (eBioscience Inc.; Thermo Fisher Scientific, Waltham, MA, USA) (36). In summary, 96-well plates were first coated overnight in a refrigerator mixed with an immunoaffinity-purified polyclonal sheep antibody specifically for each cytokine. Plates were then blocked for 2 h. Subsequently, recombinant murine TNF-α and IL-1β standards were added to the plates using a serial dilution protocol for the standard curve. For sample testing, 100 μL of samples in duplicate were added and incubated for 1 h at room temperature followed by the addition of rabbit biotinylated immunoaffinity-purified antibodies anti-TNF-α and anti-IL-1β and incubation for 1 h at room temperature. In the next step, 50 μL of avidin-HRP reagent were added to each well in a dilution of 1:5000 for 30 min of incubation at room temperature. Plates were then washed, and the o-phenylenediamine dihydrochloride substrate was added to the plates at a volume of 200 μL per well to produce measurable signals. Finally, after 15 min, the reaction was interrupted with 1 M H_2_SO_4_ and measured spectrophotometrically at 450 nm. The detection limit of TNF-α (cat. no. 147325) and IL-1β (cat. no. 147012) kits is 8 picograms (pg)/milliliter (mL). The results were expressed as pg of target cytokine per mL of plasma (32).

### Reverse Transcription and Quantitative Polymerase Chain Reaction (RT-qPCR) Assay

RT-qPCR was carried out following the protocol as described previously (32). Spinal cord (L_4_-L_6_ entire segments) and DRG (bilateral L_4_-L_6_) samples were collected using TRIzol™ reagent (#15596026, Invitrogen; Thermo Fisher Scientific, Waltham, MA, USA) 7–28 days after the infection, depending on the experiment. The purity of total RNA was measured with a spectrophotometer (MultiSkan GO Microplate Spectrophotometer, ThermoScientific, Vantaa, Finland), and the wavelength absorption ratio (260/280 nm) was between 1.8 and 2.0 for all preparations. Reverse transcription of total RNA to cDNA and qPCR were performed using the Go Taq^®^ 2-Step RT-qPCR system (Promega Corporation, Madison, WI, USA) according to the manufacturer’s guidelines. The relative gene expression was measured using the comparative 2^-(ΔΔCq)^ method. The expression of *β-actin* RNA was used as a reference gene to normalize data. The mouse primer pairs used were as follows:

*β-actin* fwd: 5’-AGCTGCGTTTTACACCCTTT-3’
*β-actin* rev: 5’-AAGCCATGCCAATGTTGTCT-3’
*Gfap* fwd: 5´-GCGCTCAATGCTGGCTTCA-3´
*Gfap* rev: 5´-TCTGCCTCCAGCCTCAGGTT-3´
*Iba1* fwd: 5´-TGGAGTTTGATCTGAATGGAAAT-3´
*Iba1* rev: 5´-CAGGGCAGCTCGGAGATAGCTTT-3´
*Cx3cr1* fwd: 5´-CACCATTAGTCTGGGCGTCT-3´
*Cx3cr1 rev*: 5´-GATGCGGAAGTAGCAAAAGC-3´
*Tnfα* fwd: 5´-TCTCATCAGTTCTATGGCCC-3´
*Tnfα* fwd: 5´-GGGAGTAGACAAGGTACAAC-3´
*Il1β* fwd: 5′-GAAATGCCACCTTTTGACAGTG-3′
*Il1β* rev: 5′-TGGATGCTCTCATCAGGACAG-3′
*Cx3cl1* fwd: 5´-ATTGGAAGACCTTGCTTTGG-3´
*Cx3cl1* rev: 5´-GCCTCGGAAGTTGAGAGAGA-3´
*Cox2* fwd: 5´-GTGGAAAAACCTCGTCCAGA-3´
*Cox2* rev: 5´-GCTCGGCTTCCAGTATTGAG-3´
*Trpv1* fwd: 5´- TTCCTGCAGAAGAGCAAGAAGC-3´
*Trpv1* rev: 5´-CCCATTGTGCAGATTGAGCAT-3´

### Western Blot Assay

Western blot analyses of the spinal cords were processed according to the previous study by our group (32). L_4_–L_6_ entire segments of spinal cord were accurately dissected at the 7th day after the infection, and the whole samples were homogenized in radioimmunoprecipitation assay (RIPA) buffer containing a protease and phosphatase inhibitor cocktail (100X, #5872S, Cell Signaling Technology, Danvers, MA, USA). The lysates were then homogenized and centrifuged. The protein extracts were separated by SDS-PAGE and transferred onto a nitrocellulose membrane (GE Healthcare-Amersham, Pittsburgh, PA, USA). Membranes were then incubated in blocking buffer 95% nonfat milk in Tris-buffered saline with Tween 20 or 1% bovine serum albumin (BSA) for different times for each antibody at 4°C in the presence of the primary antibody. β-actin and GFAP were purchased from Cell Signaling Technology (Danvers, MA, USA). Iba-1 and the secondary antibody were purchased from Thermo Fischer Scientific (Waltham, MA, USA). Catalog numbers of products are indicated below. The antibodies and dilutions used were β-actin (#4970, Cell Signaling Technology, 1:1000) and blocked with 5% BSA; GFAP (#12389, Cell Signaling Technology, 1:10,000) on 8% gel and blocked with 5% BSA; and Iba-1 (#16-20001, Wako Chemicals, 1:800) on 15% gel and blocked with 5% BSA. The molecular masses of protein were confirmed by Precision Plus Protein Standards (Bio-Rad, Hercules, CA, USA). After washing in tris buffered saline (TBS) with Tween 20, the membrane was incubated with secondary antibody (peroxidase-conjugated AffiniPure goat anti-rabbit IgG (H+L), #111-035-003, Jackson Immuno Research, 1:5000) on 5% BSA in TBS-T for 2 h at room temperature. Protein was visualized by chemiluminescence with an enhanced chemiluminescence ECL detection reagent (Luminata™ Forte, Millipore, USA). The membranes were stripped and re-probed with an antibody against β-actin for use as a loading control in addition to loading the same amount of protein in the case of Iba-1 protein. For GFAP protein analysis, a separate membrane using an equivalent mirror gel was prepared for the quantification of β-actin because the chemiluminescent signal for GFAP was still detected after the stripping phase as the stripping buffer failed to completely remove the GFAP antibody and ECL reagent from the membrane in the tested dilutions (dilution between 1:1000 and 1:10,000). Results are presented as the mean of six animals per group and compare noninfected and *T. cruzi*–infected groups at each time point (7–14 days) because experiments were performed separately. The images and analyses were performed in Image Lab 6.1 software (Bio-Rad Laboratories).

### Spinal Cord Immunofluorescence

The spinal cord immunofluorescence assay was performed at the 7th day of infection. For this, mice were perfused through the ascending aorta with phosphate buffered saline (PBS) followed by 4% of paraformaldehyde (PFA), and L_4_–L_6_ segments of the spinal cord were accurately dissected out and postfixed in PFA 4% for 24 h. After this period, samples were replaced with 30% saccharose solution for 3 additional days. The spinal cord segments were then washed with PBS and embedded in optimum cutting temperature (O.C.T.) using Tissue-Tek^®^ reagent (Sakura^®^ Finetek USA, Torrance, CA), and 10-micrometers (μm) sections were cut in a cryostat (CM1520, Leica Biosystem, Richmond, IL, USA) and processed for immunofluorescence (four slides per mouse/four animals per group). All the sections were initially blocked with a buffer solution (500 μL per slide containing PBS plus 0.1% tween 20 plus 5% BSA) for 2 h at room temperature and subsequently incubated overnight at -4°C with a solution containing primary antibodies against the target protein. Next, a new incubation with secondary antibodies was performed for 1 h at room temperature. For double immunofluorescence, a mixture of primary antibodies for target proteins as well as secondary conjugated antibodies following the same sequence was performed. GFAP (#180063; 1:500 dilution; Invitrogen, Life Technologies, Carlsbad, CA, USA), Iba-1 (#PA5-27436; 1:500 dilution, Invitrogen, Life Technologies, Carlsbad, CA, USA), and phosphorylated NFκB p65 subunit (sc-136,548, 1:200 dilution, Santa Cruz Biotechnology, Dallas, TX, USA) primary antibodies, and Alexa Fluor 488 (#A-110088, 1:1000 dilution, Thermo Fischer Scientific, Waltham, MA, USA) and Alexa Fluor 647 (#A28181, 1:1000 dilution, Thermo Fischer Scientific, Waltham, MA, USA) secondary antibodies were used in the study. The slide assembly was conducted using ProLong™ Gold Antifade Mountant with 4’,6-diamidino-2-phenylindole, dihydrochloride (DAPI) melting media (#P36931, Thermo Fischer Scientific, Waltham, MA, USA). Analysis using slides with secondary antibodies alone was conducted in parallel with the controls to ensure that unspecified staining did not occur. Immunofluorescence analyses were performed in the dorsal horn of the spinal cord at a magnification of 20x. The images and analysis were performed using a confocal microscope (TSC SP8, Leica Microsystems, Mannheim, Germany) (32, 37). Results of the fluorescence quantification in the samples (control noninfected versus 7-day infected animals) are presented as the mean of four animals per group.

### NFκB Activation Test

The evaluation of NFκB activation in spinal cord samples was performed following the protocol as described previously (32). For this objective, spinal cord samples (L_4_–L_6_ entire segments) were collected at the 7th day after the infection and the whole sample homogenized in ice-cold lysis buffer (Cell Signaling Technology, Beverly, MA, USA). The homogenates were centrifuged for 10 min (16,000 g and 4°C), and the resultant supernatants used to assess the levels of total and phosphorylated (p) NFκB p65 subunits by ELISA using two PathScan kits (Cell Signaling Technology, Beverly, MA, USA) specific for total and phosphorylated forms of NFκB p65 according to the manufacturer’s instructions. The test indicates the proportion between total NF-kB and p-NF-kB in the analyzed samples. A low ratio indicates a great amount of p-NF-kB relative to total NF-kB, which indicates NF-kB activation. On the other hand, a high ratio indicates lessened activation because there is less p-NF-kB relative to total NF-kB. The results were expressed as a total-p65/phospho-p65 ratio measured spectrophotometrically (MultiSkan GO Microplate Spectrophotometer, ThermoScientific, Vantaa, Finland).

### Calcium Imaging

The dissection and culture of DRG neurons for calcium imaging was performed as previously described (38). Bilateral DRGs (six mice per group) were dissected at the 7th day p.i. into neurobasal‐A medium (Life Technologies, Thermo Fisher Scientific) and dissociated in collagenase A (1 mg·ml−1)/dispase II (2.4 U·ml−1; RocheApplied Sciences, Indianapolis, IN, USA) in HEPES‐buffered saline (Millipore Sigma) for 70 min at 37°C. After trituration with glass Pasteur pipettes of decreasing size, DRG cells were centrifuged over a 10% BSA gradient and plated on laminin‐coated cell culture dishes. DRGs were then loaded with 1.2 μM of Fluo‐4AM in neurobasal‐A medium, incubated for 30 min at 37°C, washed with HBSS, and imaged in a Confocal Microscope (TCS SP8, Leica Microsystems, Mannheim, Germany). To assess TRPV1 activation, DRG plates were recorded for 6 min, which was divided into 2 min of initial reading (0‐s mark, baseline values), followed by stimulation with TRPV1 agonist capsaicin for 2 min at the 120‐s mark (1 μM) and KCl for 2 min at the 240‐s mark (40 mM, activates all neurons). Calcium flux was analyzed from the mean fluorescence intensity measured with the LAS X Software (Leica Microsystems, Mannheim, Germany).

### Statistical Analysis

Results are presented as means ± SEM of measurements made on four to six mice in each group, depending on the analysis, per experiment, and are representative of two independent experiments. ANOVA (two-way analysis of variance) was always used for comparison between groups and different doses when responses were measured at varied times after the parasite or treatments and/or stimulus injection. Analyzed factors were treatments, time, and time vs. treatment interaction, and when interaction was significant, one-way ANOVA preceded by Tukey’s *post hoc* was performed for each time point. Differences between responses were evaluated by one-way ANOVA followed by Tukey’s *post hoc* for data of a single time point. Differences between two groups were analyzed by *t*-test. Statistical differences were considered significant when *P* < 0.05.

## Results

### Experimental *T. cruzi* Infection Induces Chronic Mechanical and Thermal Hyperalgesia Independent of Blood Parasitemia as Well as Induces a Transient Increase in Systemic TNF-α and IL-1β Levels

**Figure 1:**
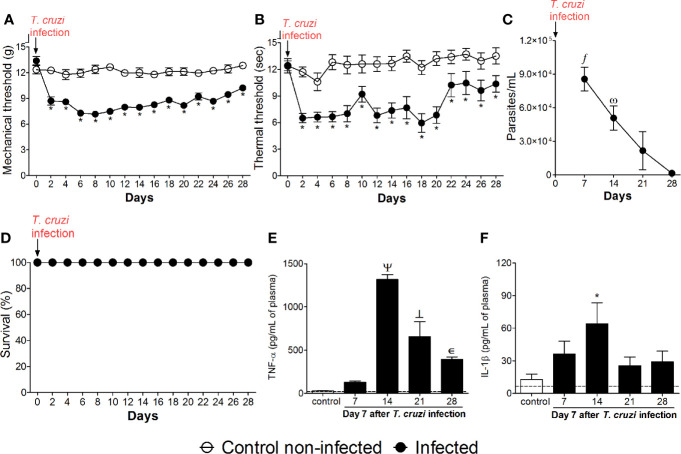
Experimental *T. cruzi* infection induces chronic pain. Mechanical hyperalgesia **(A)**, thermal hyperalgesia **(B)**, blood parasitemia **(C)**, survival **(D)**, and plasma levels of TNF-α **(E)** and IL-1β **(F)** were determined. Hyperalgesia tests were evaluated for 28 days p.i. every 2 days. Blood parasitemia and cytokine plasma levels were evaluated 7–28 days p.i. every 7 days. Survival rate was monitored daily over the model. Results are presented as mean ± SEM of six mice per group per experiment and are representative of two separated experiments. *p < 0.05 compared to control noninfected mice; *fp* < 0.05 compared to days 21 and 28; ω*p* < 0.05 compared to day 28; Ψ *p* < 0.05 compared to all groups; ┴ *p* < 0.05 compared to control noninfected mice and day 7 (two-way ANOVA followed by Tukey’s posttest for panels **(A, B)**; and one-way ANOVA followed by Tukey’s posttest for panels **(C**, **E, F)**. Dashed lines in panels **(E**, **F)** delimits the sensitivity of kits used for analysis.

Mice were infected with *T. cruzi* and mechanical hyperalgesia, thermal hyperalgesia, blood parasitemia, survival, and TNF-α and IL-1β levels were evaluated from 2 to 28 days p.i. (**Figure 1**). Increased mechanical and thermal hyperalgesia in infected mice were detected from the 2nd to the 28th days p.i. (**Figures 1A, B**). The peak of blood parasitemia occurred at the 7th day p.i. with a gradual decrease in subsequent days, and at the 28th day, the parasites were no longer detected in the blood (**Figure 1C**). Interestingly, in infected mice, the hyperalgesia persists even with the decline of parasites in the blood (**Figures 1A, B**), suggesting that not only the parasites per se are responsible for the pain behavior, but possibly plastic changes are involved in the prolonged hyperalgesia. No animals died during the experiment due to *T. cruzi* infection, which is an expected outcome due to the use of a nonlethal parasite load for the C57BL/6 mouse strain (**Figure 1D**). TNF-α plasma levels were not significant at the 7th day p.i. (**Figure 1E**). TNF-α plasma levels were significant at the 14th day with a gradual reduction from the 14th day onward; however, they remained significant until the 28th day p.i. compared to uninfected animals (**Figure 1E**). IL-1β plasma levels were significant at the 14th day without significant levels in the other time points (**Figure 1F**). Altogether, these results indicate that *T. cruzi* infection at the tested dose induces chronic pain. However, factors other than solely blood parasite load and circulating levels of TNF-α and IL-1β might explain *T. cruzi* infection-induced pain overtime.

### Experimental *T. cruzi* Infection Activates Astrocytes and Microglia in the Spinal Cord

**Figure 2:**
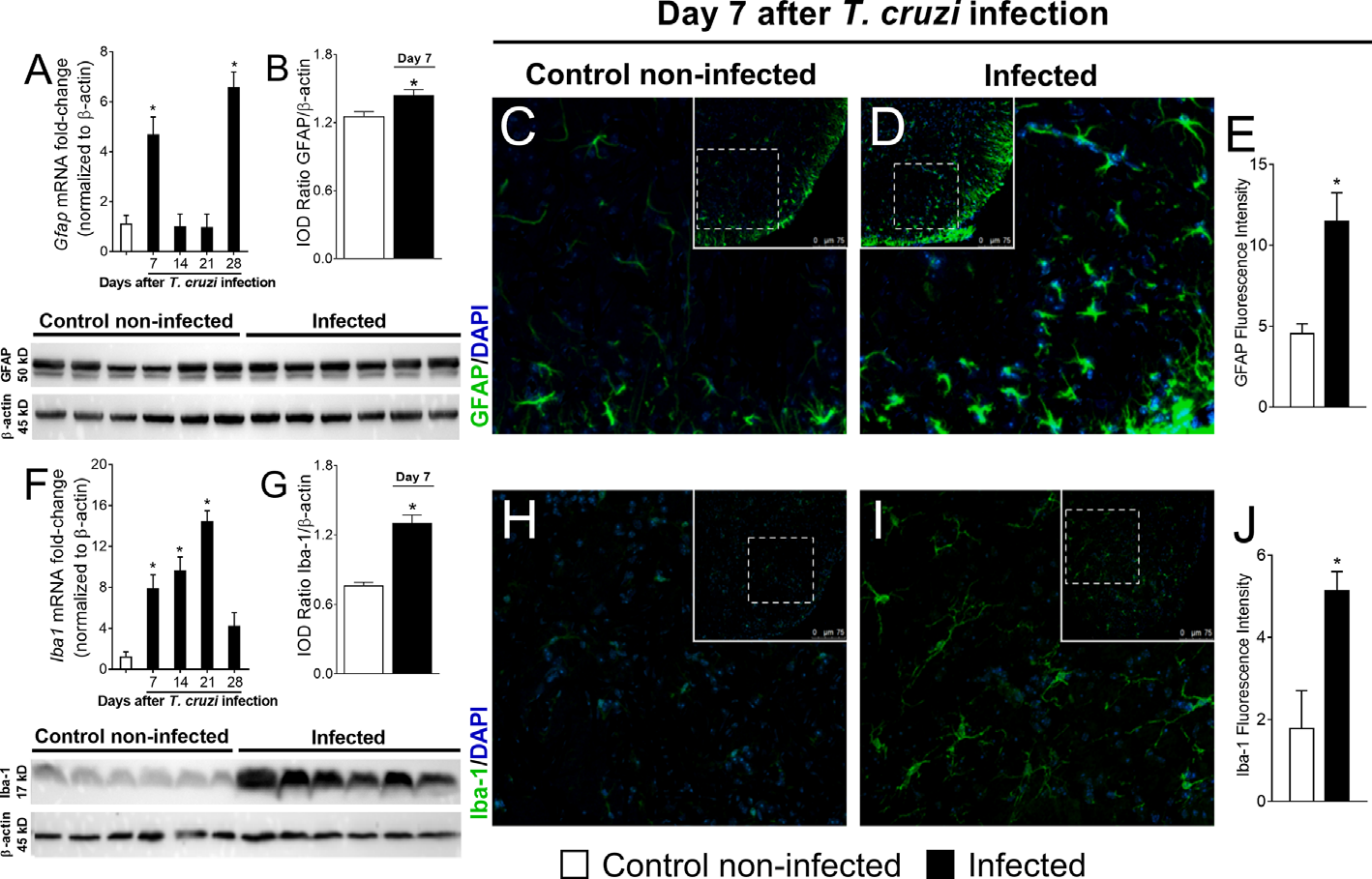
Experimental *T. cruzi* infection induces spinal cord astrocytes **(A–E)** and microglial **(F–J)** activation. *Gfap* and *Iba1* mRNA expression was determined in control noninfected and infected mice 7–28 days p.i. every 7 days by RT-qPCR **(A, F**, respectively). At day 7 p.i. (peak of *Gfap* and *Iba1* mRNA expression), Western blot analysis of the spinal cord was performed to confirm GFAP **(B)** and Iba-1 **(G)** expression. Next, 7-day spinal cord samples were stained with antibodies for astrocytes **(C–E)** and microglia **(H–J)** (GFAP and Iba-1, respectively; green) and regular nucleus (DAPI, blue) detection. Representative immunostainings of the spinal cord of control noninfected and infected mice are shown in panels **(C, D, H, I)**, respectively (20x magnification, scale bar 75 μm with zoom). Panels **(E, J)** show the percentage of GFAP and Iba-1 fluorescence intensity in each experimental group, respectively. Results are presented as mean ± SEM of six **(A, B, F, G)** or four mice **(E, J)** per group per experiment and are representative of two separate experiments. *p < 0.05 compared to control noninfected mice (one-way ANOVA followed by Tukey’s posttest).

We evaluated whether *T. cruzi* infection activates glial cells in the spinal cord of mice. First, we determined the temporal profile of *Gfap* and *Iba1* mRNA expression (7–28 days p.i.; **Figures 2A–F**), which are used as markers of astrocytes and microglial activation in the spinal cord, respectively. The activation profile of these two cells did not occur in a similar manner. *Gfap* mRNA presented a biphasic increase, and for *Iba1* mRNA time-dependent upregulation was observed until the 21st day p.i., when it reached its peak (**Figures 2A, F**). At the 7th day, infected mice presented increased *Gfap* and *Iba1* expression when compared to uninfected mice. However, thenceforward *Gfap* expression returned to the control level at days 14 and 21 p.i. and increased again at day 28 (**Figure 2A**). Instead, *Iba1* mRNA expression presented a sustained upregulation until the 21st day p.i., decreasing and ceasing to be significant compared to uninfected animals at day 28 p.i. (**Figure 2F**). A time course of GFAP and Iba-1 protein levels was also performed by Western blot analysis (**Supplementary Figure S1**), which showed that GFAP was significantly upregulated in the infected group at the 7th day (**Figures 2 B–G**). GFAP was also upregulated at days 21 and 28, thus, partially aligning with the mRNA data (**Supplementary Figure S1A**). A contrast between mRNA and protein data for GFAP was observed at the 21st day in which mRNA was not induced, but protein was increased (**Supplementary Figure S1A**). Iba-1 protein was increased in the infection group at all time points (**Supplementary Figure S1B**) contrasting with the mRNA data in which, at the 28th day, no upregulation was observed in the infected group (**Figure 2F**). An immunofluorescence assay in spinal cord samples for GFAP and Iba-1 was also conducted and showed a classical morphology of activated astrocytes and microglia in infected animals 7 days p.i., which was not observed in uninfected animals (**Figures 2C–E, H–J**, respectively). Time-response analysis (7–28) of GFAP and Iba-1 immunofluorescence detected significant activation of both glial cells in infected animals when compared to those uninfected at all evaluated times (**Supplementary Figures S2A, B**). Considering that concomitant activation of astrocytes and microglia was observed at the 7th day p.i. for all tests used (RT-qPCR, Western blot, and immunofluorescence), this time point was chosen for the next experiments to focus on the contribution of spinal cord glial cells to *T. cruzi* infection-induced pain in mice. These methods (RT-qPCR, Western blot, and immunofluorescence) quantitate different parameters (mRNA vs. protein), and the methods’ sensibilities are different, which might explain the variations observed and that mRNA and protein data did not exactly align in the same way.

### Spinal Treatment With Glial Inhibitors Reduces *T. cruzi*–Induced Mechanical and Thermal Hyperalgesia

**Figure 3:**
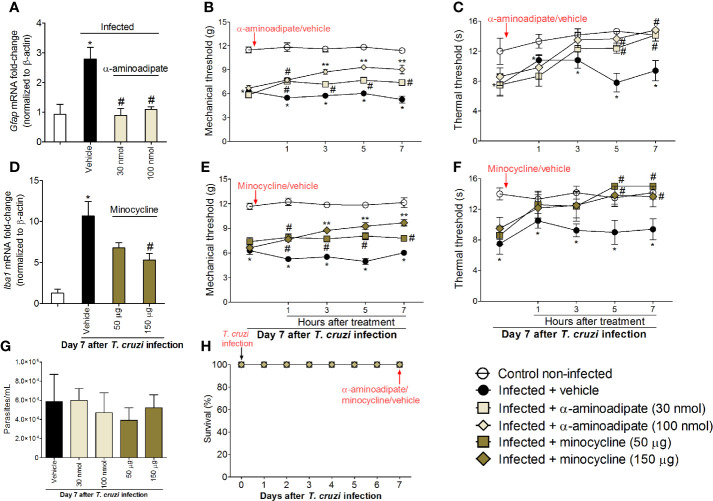
Targeting spinal cord glial cells with α-aminoadipate and minocycline intrathecal treatments inhibits *T. cruzi*–induced hyperalgesia. Panels **(A–C)** show RT-qPCR data for *Gfap* mRNA expression and mechanical and thermal hyperalgesia after vehicle and α-aminoadipate treatments (30 and 100 nmol) at the 7th day p.i. Mensuration of hyperalgesia occurred 1–7 h after the treatments. Panels **(D–F)** show RT-qPCR data for *Iba1* mRNA expression and mechanical and thermal hyperalgesia after vehicle and minocycline treatments (50 and 150 μg) at the 7th day p.i. Panel **G** presents blood parasitemia at the 7th day p.i. 7 h after the treatments. Panel **H** presents survival rates during the experimental protocol. Results are presented as mean ± SEM of six mice per group per experiment and are representative of two separate experiments. *p < 0.05 compared to control noninfected mice; ^#^ *p* < 0.05 compared to infected mice treated with vehicle; **p < 0.05 compared to infected mice treated with the lowest doses of α-aminoadipate and minocycline (one-way ANOVA followed by Tukey’s posttest for panels **A**, **D**; and two-way ANOVA followed by followed by Tukey’s posttest for panels **B**, **C**, **E**, **F**).

To investigate whether inhibiting spinal cord glial cells reduces *T. cruzi*–induced hyperalgesia, mice were treated by the intrathecal route with vehicle (saline); a selective astrocyte inhibitor, α-aminoadipate (30–100 nmol); or a selective microglial inhibitor, minocycline (50–150 μg), at day 7 after *T. cruzi* infection, and mechanical and thermal hyperalgesia were measured 1–7 h after the treatment (**Figure 3**). To validate α-aminoadipate and minocycline effects, mRNA expression of *Gfap* and *Iba1* were also evaluated at the 7th day p.i. The two doses of α-aminoadipate inhibited *Gfap* mRNA expression; on the other hand, only the highest dose of minocycline inhibited *Iba1* mRNA expression (**Figures 3A–D**). The doses of 30 and 100 nmol of α-aminoadipate inhibited mechanical hyperalgesia for up to 7 h after the treatment; however, the effect of the dose of 100 nmol was significantly higher than 30 nm between 3 and 7 h (**Figure 3B**). On thermal hyperalgesia, both doses of α-aminoadipate presented similar activity and inhibited thermal sensitivity at 5 and 7 h after treatment (**Figure 3C**). A similar profile was observed by the treatment with the microglia selective inhibitor, minocycline, regarding mechanical (**Figure 3E**) and thermal (**Figure 3F**) hyperalgesia. The fact that the lowest dose of minocycline did not inhibit the mRNA expression of Iba-1 in the spinal cord but inhibited hyperalgesia can be explained by the fact that Iba-1 or even GFAP are used as markers of glial cell activation and are not necessarily activity measures. Further, α-aminoadipate and minocycline are inhibitors of glial cell metabolism (39, 40), and it is reasonable that a higher dose will have faster activity than a lower dose as well as that makers of activation might change after actual downregulation of activity. Intrathecal treatments with the tested doses of glial inhibitors did not affect blood parasitemia (**Figure 3G**), and no animal died due to infection or treatment protocols (**Figure 3H**). These data corroborate the results presented in **Figure 2** and reinforce the concept of a role for spinal cord astrocytes and microglia in *T. cruzi*–induced hyperalgesia.

### Targeting Spinal Cord NFκB Inhibits *T. cruzi*–Induced Glial Activation and Hyperalgesia as Well as Glial Inhibitors Reduce NFκB Activation in the Spinal Cord

**Figure 4:**
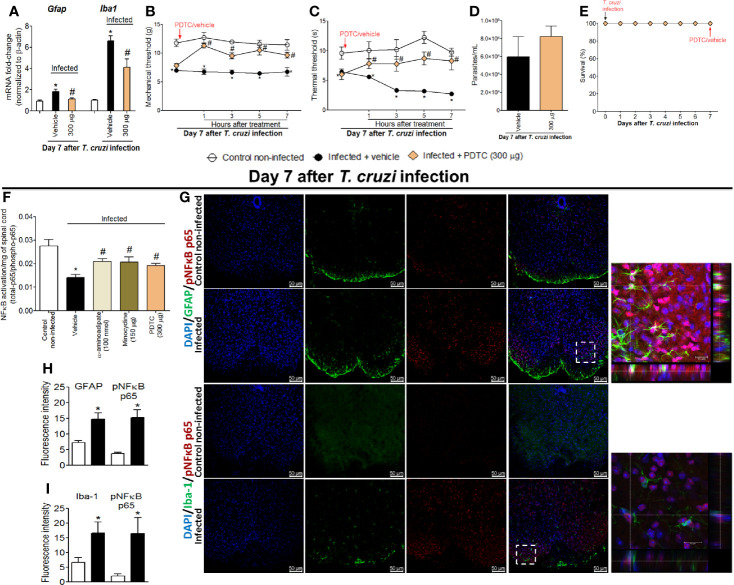
Targeting spinal cord NFκB with PDTC intrathecal treatment inhibits *T. cruzi*–induced glial activation and hyperalgesia as well as targeting glial cells with α-aminoadipate and minocycline reduced spinal cord NFκB activation. Panels **(A–C)** show RT-qPCR data for *Gfap* and *Iba1* mRNA expression and mechanical and thermal hyperalgesia after vehicle and PDTC treatments (300 μg) at the 7th day p.i. Panel **(D)** presents blood parasitemia at the 7th day p.i. 7 h after the treatments. Panel **(E)** presents survival rates during the experimental protocol. The effects of targeting spinal cord glial cells with α-aminoadipate and minocycline upon NFκB activation at the 7th day p.i. 7 h after the treatments are presented in panel **(F)**. Representative immunostainings of triple immunofluorescence (DAPI/pNFκB p65/GFAP and DAPI/pNFκB p65/Iba-1) of the spinal cord of control noninfected and infected mice are shown in **(G)** (20x magnification, scale bar 50 μm). 3-D images with zoom demonstrating pNFκB p65 staining surrounded by GFAP or Iba-1 are shown in inserts. Panels H and I demonstrate the fluorescence intensity (%) of GFAP and pNFκB p65, and Iba-1, and pNFκB p65, respectively. Results are presented as mean ± SEM of six **(A–F)** or four mice **(G–I)** per group per experiment and are representative of two separate experiments. *p < 0.05 compared to control noninfected mice; ^#^ *p* < 0.05 compared to infected mice treated with vehicle (one-way ANOVA followed by Tukey’s posttest for panels **(A**, **F)**; and two-way ANOVA followed by followed by Tukey’s posttest for panels **B**, **C**).

The participation of the transcription factor NFκB in *T. cruzi*–induced spinal cord glial activation and hyperalgesia was evaluated. Mice were treated by the intrathecal route with vehicle (saline) or NFκB inhibitor PDTC (300 μg) at day 7 after *T. cruzi* infection, and mRNA expression of glial markers and mechanical and thermal hyperalgesia were measured. Additionally, the effects of glial inhibitors upon spinal cord NFκB activation and double staining using the phosphorylated (p) NFκB p65 subunit and glial markers in spinal cord samples were also performed (**Figure 4**). PDTC inhibited the increased expression of *Gfap* and *Iba1* in infected animals (**Figure 4A**) as well as the mechanical and thermal hyperalgesia at all time points evaluated (1–7 h) upon *T. cruzi* infection (**Figures 4B, C**). PDTC treatment did not affect blood parasitemia (**Figure 4D**). Furthermore, no animal died due to infection or treatment protocols (**Figure 4E**). Extending these experiments, we notice that the most effective doses of glial inhibitors (100 nmol of α-aminoadipate and 150 μg of minocycline) inhibited *T. cruzi*–induced NFκB activation in the spinal cord (demonstrated here by the ratio between total p65 subunits per phosphorylated p65 subunit), reaching similar levels of inhibition as positive control PDTC (**Figure 4F**). Finally, using a confocal immunofluorescence assay, we showed the concomitant expression of GFAP (**Figure 4G**, two top rows) or Iba-1 (**Figure 4G**, two lower rows) and phosphorylated pNFκB p65 (**Figure 4G**, last two columns on the right and respective inserts) in infected animals, which was not observed in uninfected mice (**Figure 4G**). As GFAP and Iba-1 are cell membrane molecules, they do not co-localize with NFκB, which has cytoplasmic or, in case of phosphorylation, nuclear localization. The 3-D images (inserts in **Figure 4G**) and videos (**Videos S1** and **S2**) allow visualization that part of pNFκB is surrounded by GFAP and Iba-1 staining. Further supporting that NFκB activation aligns with glial cell activation, increased fluorescence intensity of glial markers and pNFκB were detected in infected animals when compared to those uninfected (**Figures 4H, I**). Therefore, these results evidence that *T. cruzi* infection induces NFκB activation in spinal cord astrocytes and microglia, contributing to hyperalgesia as a result of the disease.

### Temporal Profile of Spinal Cord *Cx3cr1*, *Tnfα*, and *Il1β* Expression in Response to Experimental *T. cruzi* Infection

**Figure 5:**
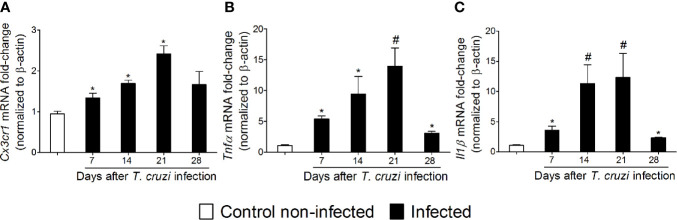
Experimental *T. cruzi* infection induces spinal cord C*x3cr1* **(A),** *Tnfα* **(B)**, and *Il1β* **(C)** mRNA expression. Time-course expressions of mRNA were evaluated 7–28 days p.i. every 7 days. Results are presented as mean ± SEM of six mice per group per experiment and are representative of two separate experiments. *p < 0.05 compared to control noninfected mice; #*p* < 0.05 compared to infected mice treated with vehicle (one-way ANOVA followed by Tukey’s posttest).

NFκB is a central transcription factor in regulating cytokine production as they are important signaling molecules in neuro-immune regulation and pain (17). The chemokine receptor and cytokine mRNA expression profile were investigated between 7 and 28 days after *T. cruzi* infection (**Figure 5**). *T. cruzi* infection induced a progressive increase in mRNA expression of the chemokine receptor *Cx3cr1* (**Figure 5A**) and pro-inflammatory/hyperalgesic cytokines *Tnfα* (**Figure 5B**) and *Il1β* (**Figure 5C**) until the 21st day p.i. with a statistical difference compared to those uninfected. After this period, their expression declined and returned close to the baseline values (day 28) (**Figures 5A–C**). These data, together with the glial and NFκB activation (Figs. 2–4), depict spinal cord neuroinflammation in response to *T. cruzi* infection. Microglia is the main cell expressing CX_3_CR1 in the spinal cord, and both astrocytes and microglia are well known sources of cytokines in the spinal cord (17).

### Targeting Spinal Cord CX_3_CL1, TNF-α, and IL-1β Reduces *T. cruzi*–Induced Mechanical and Thermal Hyperalgesia

**Figure 6:**
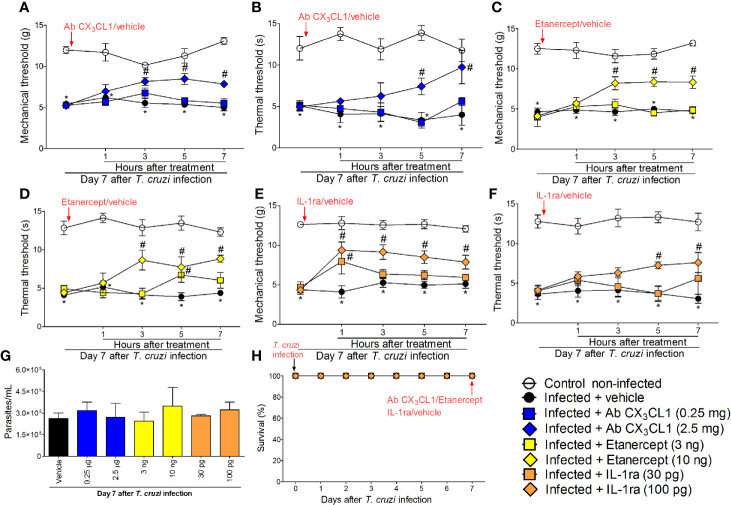
Targeting spinal cord CX_3_CL1, TNF-α, and IL-1β with neutralizing antibody anti-CX_3_CL1, etanercept, and IL-1ra intrathecal treatments, respectively, inhibits *T. cruzi*–induced hyperalgesia. Mechanical hyperalgesia **(A, C, E)** and thermal hyperalgesia **(B, D, F)** were evaluated at the 7th day p.i., after vehicle, neutralizing antibody anti-CX_3_CL1 (0.25 and 2.5 mg), etanercept (3 and 10 ng), and IL-1ra (30 and 300 pg) intrathecal treatments, 1–7 h after the treatments. Panel **(G)** presents blood parasitemia at the 7th day p.i. 7 h after the treatments. Panel **(H)** presents survival rates during the experimental protocol. Results are presented as mean ± SEM of six mice per group per experiment and are representative of two separate experiments. *p < 0.05 compared to control non-infected mice; ^#^ *p* < 0.05 compared to infected mice treated with vehicle (two-way ANOVA followed by followed by Tukey’s posttest).

In addition to the mRNA expression data, we wanted to ascertain the physiopathological contribution of spinal cord CX_3_CL1/CX_3_CR1 signaling, TNF-α, and IL-1β in *T. cruzi*–induced hyperalgesia. For this purpose, mice were treated by the intrathecal route with vehicle (saline), anti-CX_3_CL1 antibody (0.25–2.5 mg), soluble decoy receptor (sTNFR2) etanercept (3-10 ng) and Interleukin-1 receptor antagonist (IL-1ra, 30-100 pg) at day 7 after *T. cruzi* infection. Mechanical and thermal hyperalgesia were measured 1–7 h after the treatment (**Figure 6**). The dose of 0.25 mg of anti-CX_3_CL1 antibody did not affect mechanical or thermal hyperalgesia; however, treatment with the dose of 2.5 mg inhibited mechanical hyperalgesia between 3 and 7 h and thermal hyperalgesia between 5 and 7 h after the treatment (**Figures 6A, B**). The dose of 3 ng of etanercept inhibited only thermal hyperalgesia at 5 h without presenting effects on mechanical hyperalgesia. On the other hand, treatment with the dose of 10 ng of etanercept inhibited both mechanical and thermal hyperalgesia from 3 to 7 h after the treatment (**Figures 6C, D**). Treatment with the dose of 30 pg of IL-1ra inhibited only mechanical hyperalgesia at 1 h without showing effects upon thermal hyperalgesia, and the anti-hyperalgesic effect of the dose of 100 pg of IL-1ra was detected between 1 and 7 for mechanical hyperalgesia and between 5 and 7 for thermal hyperalgesia (**Figures 6E, F**). All doses of the compounds tested in these experiments did not affect blood parasitemia (**Figure 6G**), and no animal died as a result of infection or treatment protocols (**Figure 6H**). These data (**Figure 6**) line up well with the mRNA expression results presented in **Figure 5** and demonstrate a role for CX_3_CL1/CX_3_CR1 signaling, TNF-α, and IL-1β in hyperalgesia induced by *T. cruzi* infection.

### Targeting Spinal Cord Astrocytes, Microglia, and NFκB Inhibits *Cx3cr1*, *Tnfα*, and *Il1β* Expression in the Spinal Cord

**Figure 7:**
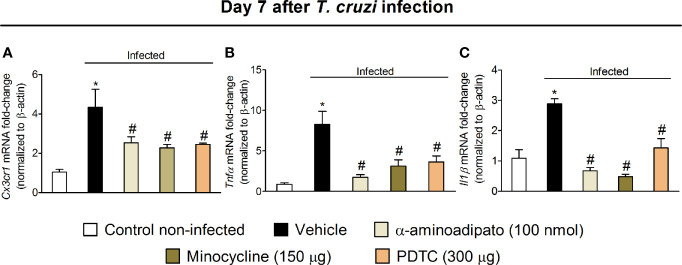
Targeting spinal cord glial cells and NFκB with α-aminoadipate, minocycline, and PDTC intrathecal treatment inhibits *T. cruzi*–induced spinal cord C*x3cr1* **(A),** *Tnfα* **(B)**, and *Il1β* **(C)** increased mRNA expression. Evaluations were performed at the 7th day p.i. after vehicle, α-aminoadipate (100 nmol), minocycline (150 μg), and PDTC (300 μg) intrathecal treatments, 7 h after the treatments. Results are presented as mean ± SEM of six mice per group per experiment and are representative of two separate experiments. *p < 0.05 compared to control noninfected mice; ^#^ *p* < 0.05 compared to infected mice treated with vehicle (one-way ANOVA followed by Tukey’s posttest).

The results of Figs. 1–6 show that *T. cruzi* infection induces pain that is amenable by inhibition of spinal cord glial cells, NFκB, chemokines, and cytokines and that the activation of glial cells is NFκB dependent. In this section, we addressed whether chemokine and cytokine expression would be dependent on NFκB and glial cells (**Figure 7**). Mice were treated by the intrathecal route with vehicle (saline) or the most effective dose of each inhibitor tested earlier, which include 100 nmol of α-aminoadipate, 150 μg minocycline, and 300 μg of PDTC. Treatments with all inhibitors significantly reduced *T. cruzi*–induced mRNA expression of *Cx3cr1* (**Figure 7A**), *Tnfα* (**Figure 7B**), and *Il1β* (**Figure 7C**) in the spinal cord. These data corroborate the results presented in Figs. 3–6 and indicate that spinal cord astrocytes, microglia, and NFκB activation are responsible for the production of major molecules related to the sensitization and activation process of nociceptor sensory neurons in *T. cruzi* infection.

### Experimental *T. cruzi* Infection Increases Activation of DRG Neurons

**Figure 8:**
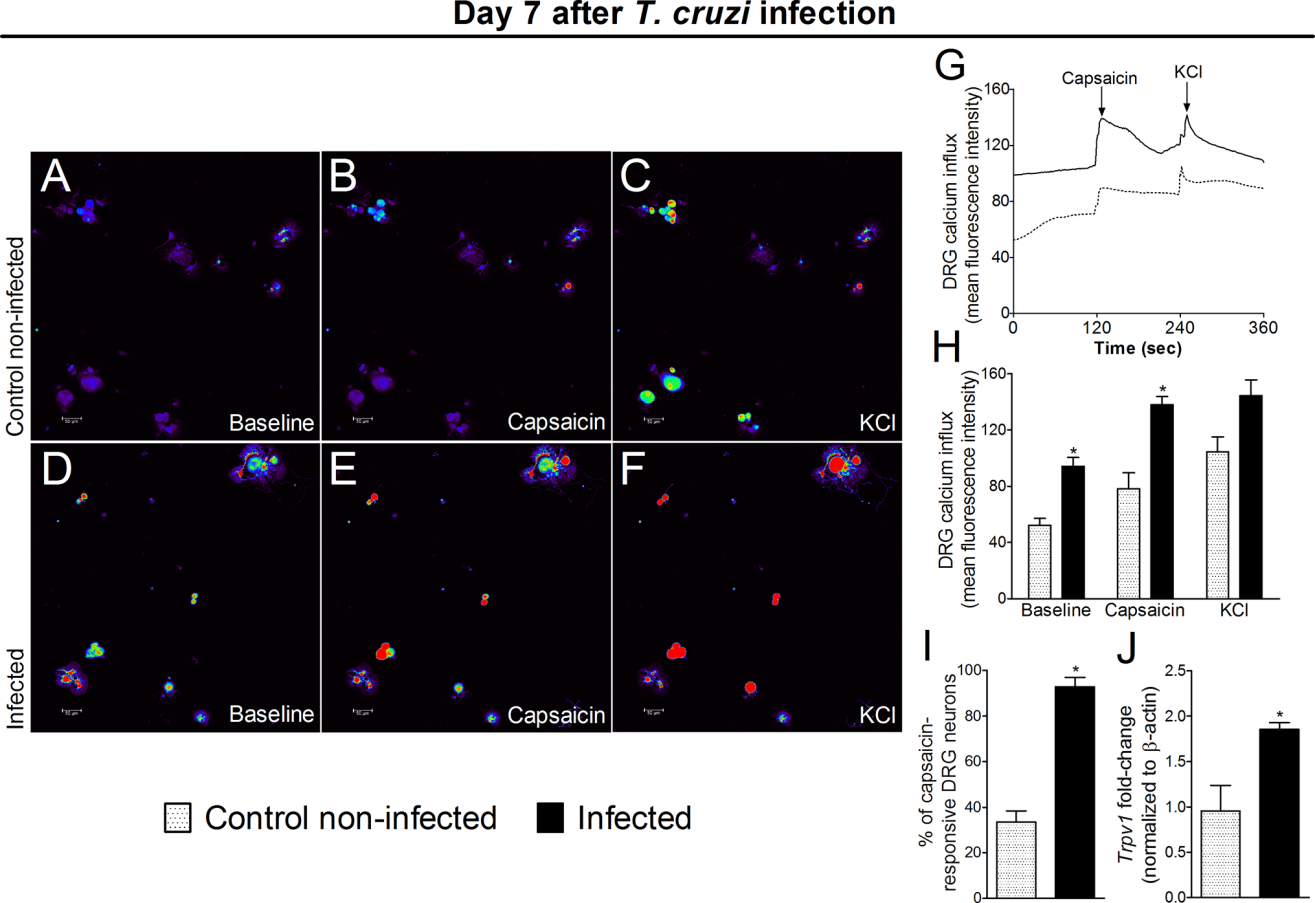
Experimental *T. cruzi* infection induces the activation of DRG neurons. Seven days after the infection, DRGs were dissected for calcium imaging using Fluo‐4AM **(A**–**I)** and mRNA expression by RT‐qPCR **(J)**. Panels **(A**–**F)** display representative fields of DRG neurons from control noninfected **(A, C)** and infected **(D–F)** mice. Panels **(A, D)**: baseline fluorescence (first column); panels **(B, E)** fluorescence after capsaicin (second column); and panels **(C, F)** after KCl control (third column). Panel **G** displays the mean fluorescence intensity traces of calcium influx from the representative DRG fields **(A**–**F)** throughout the 6 min of recording. The representative traces show that DRG neurons of infected mice presented higher calcium levels in the baseline than those DRG neurons of control noninfected mice. Panel **(H)** shows the mean fluorescence intensity of calcium influx of the baseline (0‐s mark) and that following the stimulus, either capsaicin (120‐s mark, TRPV1 agonist) or KCl (240‐s mark, activates all neurons). Panels **(I**, **J)** shows the capsaicin-responsive DRG cells and RT‐qPCR data, demonstrating that infected mice present an increased percentage of responsive cells and *Trpv1* mRNA expression, respectively. Results are expressed as mean ± SEM; *n* = 4 DRG plates (each plate is a neuronal culture pooled from six mice) per group per experiment, and RT‐qPCR used *n* = 6 DRG per group per experiment and are representative of two separate experiments. *p < 0.05 compared to control noninfected mice (one‐way ANOVA followed by Tukey’s posttest).

The response of DRG neurons to experimental *T. cruzi* infection was evaluated through calcium imaging and *Trpv1* mRNA expression (an ion channel involved in nociceptor neuron activation). DRG neurons from infected mice presented a higher baseline level of calcium influx as well as a higher response to capsaicin than DRG neurons of uninfected mice (**Figures 8A–H**). In addition to a higher calcium baseline, DRG neurons of infected mice presented a higher intensity of response and higher percentage of responsive neurons upon capsaicin stimulation than DRG neurons of uninfected mice (**Figures 8H, I**). An explanation for a higher baseline and response to capsaicin is that DRG neurons of infected mice presented increased *Trpv1* mRNA expression compared to DRG neurons of uninfected mice (**Figure 8J**). Therefore, the behavioral responses triggered by *T. cruzi* infection parallel with activation of TRPV1-sensitive DRG neurons further confirming the nociceptive nature of behavioral responses.

### Intrathecal Treatments With Inhibitors of Astrocytes, Microglia, and NFκB Downregulates the mRNA Expression of Inflammatory Molecules in the DRG Microenvironment

**Figure 9:**
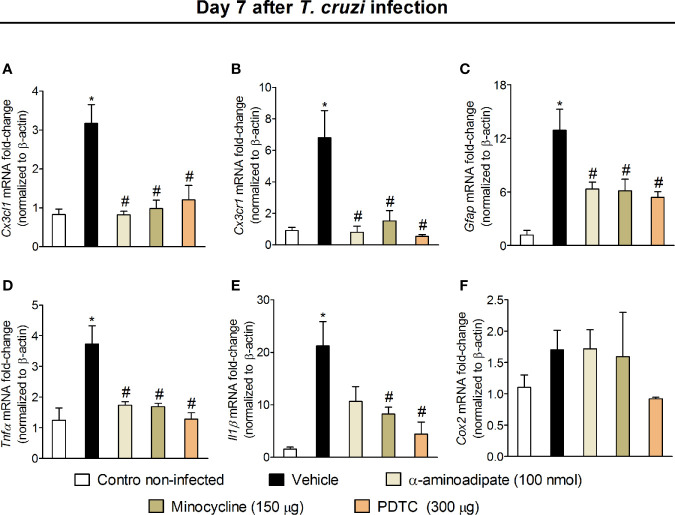
Effect of intrathecal treatment with α-aminoadipate, minocycline, and PDTC against *T. cruzi*-induced DRG C*x3cl1* **(A)**, C*x3cr1* **(B)**, *Gfap* **(C)**, *Tnfα* **(D)**, *Il1β* **(E)**, and *Cox2* **(F)** mRNA expression. Evaluations were performed at the 7th day p.i. after vehicle, α-aminoadipate (100 nmol), minocycline (150 μg), and PDTC (300 μg) intrathecal treatments, 7 h after the treatments. Results are presented as mean ± SEM of six mice per group per experiment and are representative of two separate experiments. * *p* < 0.05 compared to control noninfected mice; # *p* < 0.05 compared to infected mice treated with vehicle (one-way ANOVA followed by Tukey’s posttest).

Intrathecal treatments reach the DRG, indicating that these treatments could affect DRG cells (41) and spinal cord neuroinflammation causes retrograde nociceptor neuron sensitization (42), indicating that spinal cord treatments can affect DRG cells through neuronal signaling. Therefore, we evaluated whether intrathecal treatments with α-aminoadipate, minocycline, and PDTC would affect DRG neuroinflammation. Mice were treated by the intrathecal route with vehicle (saline) or the same doses of glial and NFκB inhibitors used in **Figure 7** experiments (100 nmol of α-aminoadipate, 150 μg minocycline, and 300 μg of PDTC). The mRNA expression for *Cx3cl1*, *Cx3cr1*, *Gfap* (marker for satellite glial cells activity), *Tnfα*, *Il1β*, and *Cox2* mRNA expression were quantitated (**Figure 9**). Intrathecal treatments targeting spinal cord astrocytes, microglia, and NFκB reduced *T. cruzi*–induced *Cx3cl1* (**Figure 9A**), *Cx3cr1* (**Figure 9B**), *Gfap* (**Figure 9C**), and *Tnfα* (**Figure 9D**) mRNA expression in DRG samples. Regarding *Il1β*, only treatments with minocycline and PDTC inhibited its mRNA expression (**Figure 9E**). There was a trend toward inhibition (*P* = 0.1236) by α-aminoadipate. This result indicates that α-aminoadipate that selectively targets astrocytes could not significantly affect *Il1β* mRNA expression. We did not observe significant changes of *Cox2* mRNA expression in the DRG (**Figure 9F**). These data indicate that intrathecal treatments targeting astrocytes, microglia, and NFκB result in reduced DRG neuroinflammation after *T. cruzi* infection.

## Discussion

The infection of resistant mouse strain C57BL/6 with a nonlethal load of *T. cruzi* resembles human acute CD (43). We show that it induces another important symptom of human CD in mice: pain (7–10). *T. cruzi* induced chronic mechanical and thermal hyperalgesia for up to 28 days p.i. The hyperalgesia could not be explained by simply correlating with blood parasite load, suggesting that plastic changes might be responsible for maintaining pain at later time points when blood parasitemia declined and/or fluctuated drastically as in the chronic phase of the infection, similarly to human CD. The investigation of changes that would explain the disease pain phenotype led to unveiling novel physiopathological mechanisms of *T. cruzi* infection-induced pain.

TNF-α and IL-1β are well-known hyperalgesic molecules that induce sensitization of nociceptors (13, 14, 17, 44, 45). High peripheral levels of these cytokines can account for the observed mechanical and thermal hyperalgesia. Recently, it was reported that bacterial infections may activate silent nociceptors *via* cytokine production (including TNF-α and IL-1β), resulting in bladder hyperalgesia (46). Thus, it is also likely that this is a contributing mechanism to *T. cruzi* infection-induced pain because there are high levels of TNFα and IL-1β in this disease (47). However, peripheral TNFα and IL-1β do not explain, alone, the peripheral component of *T. cruzi* infection-induced pain because hyperalgesia started by the 2nd day, and the production of these cytokines peaked at the 14th day of infection without significant levels at the 7th day p.i. These results raise the possibility that other peripheral cytokines/molecules might also be involved in *T. cruzi* pain. For instance, IFN-γ is a hyperalgesic cytokine (17, 48, 49). IFN-γ levels increase in the serum of mice at the 2nd day of *T. cruzi* infection (12), and spleen cells rapidly produce IFN-γ in response to *T. cruzi* (50). IL-12 is a well-known inducer of IFN-γ and also a hyperalgesic cytokine (51). IL-12 levels increase in response to *T. cruzi* in mice (52). Future studies might exhaustively investigate the mechanistic contribution of other peripheral cytokines to *T. cruzi* infection-induced pain.

We demonstrate here that experimental *T. cruzi* infection induces an early acute activation (7th day p.i.) of spinal cord astrocytes and microglia in an NFκB-dependent manner. A previous study also showed the activation of mouse brain astrocytes after *T. cruzi* (Colombian strain) infection in the acute phase of the disease (26), indicating this phenomena is not restricted to spinal cord astrocytes. Temporal differences in microglia and astrocyte activation have been reported in neuropathic and inflammatory pain (53, 54). We observed some discrepancies in the temporal profile between mRNA and protein data regarding glial cell activation. Overall, astrocyte and microglia activation could be detected at all time points by at least two techniques (RT-qPCR, Western blot, or immunofluorescence) except by GFAP at the 14th day, which was not increased in the infection group compared to the uninfected group as per RT-qPCR and Western blot results. These variations between the techniques may reflect the different sensitivity of each test for each target molecule. Additional studies that go beyond the current aims of the present work are necessary to elucidate this unique profile, its consequences, and relation to disease phenotype. In *Leishmania amazonensis* (*L. amazonensis*) infection, we observed a different profile of spinal cord glial cell activation. Both astrocytes and microglia were activated by the 30th day p.i (32). This difference is certainly explained by the characteristic pathology of each disease. *L. amazonensis* infection in BALB/c strain mice induces a chronic infection with pain up to 40 days. After this time point, there is development of ulcerating skin lesions that become painless (55). *L. amazonensis* infection presents this contrast of an initial prolonged painful phase followed by painless ulcer formation with reports of pain in other sites of the body in the case of humans (32, 56). We opted to study the spinal cord events in a CD mouse model at the 7th day p.i., considering astrocytes and microglia could be studied at the same time, reducing the number of animals.

Astrocytes, microglia, and the transcription factor NFκB are essential for hyperalgesic cytokine production in the spinal cord, contributing to central sensitization (17, 37). Indeed, *T. cruzi* infection increased spinal cord mRNA expression of *Cx3cr1*, *Tnfα*, and *Il1β*, and inhibiting spinal gliosis and NFκB activation as well as targeting NFκB-related spinal cord hyperalgesic mediators (CX_3_CL1, TNF-α, and IL-1β) reduced *T. cruzi*–induced hyperalgesia. Moreover, treatment with glial and NFκB inhibitors also counteracted the increased expression of *Cx3cr1*, *Tnfα*, and *Il1β* mRNA expression in the spinal cord. Thus, expression and functional data support the contribution of spinal cord glial cells to pain in *T. cruzi* infection *via* cytokine production. CX_3_CL1 release by nociceptor neurons in the spinal cord microenvironment acts on its receptor in microglia, leading to the production of TNF-α and IL-1β by these cells. These sequential events contribute to central sensitization (17). In addition to inducing microglia activation, CX_3_CL1 can directly mediate neuronal hyper-responsiveness (57). TNFα makes a priming of astrocytes, enhancing their susceptibility to *T. cruzi* infection creating a cycle of infection and neuroinflammation because astrocytes are producers of TNFα in the spinal cord (25). Treatment with etanercept, a soluble p75/TNFR2 receptor reduces experimental CD pain (16), further corroborating the concept that cytokines, such as TNFα, have a role in immune cell activation during CD, but also as a hyperalgesic function. IL-1β can both activate and facilitate neuronal depolarization depending on disease context and chronicity (58, 59); however, this is the first evidence that it has a hyperalgesic role in CD.

DRG neurons of *T. cruzi*–infected mice showed increased baseline levels of intracellular calcium as well as responded more effectively to the stimulation with the TRPV1 agonist capsaicin than DRG neurons of uninfected mice. The percentage of capsaicin-responsive fibers and mRNA expression of *Trpv1* in DRG neurons of infected mice were also higher than those of uninfected mice. The enhanced *Trpv1* mRNA expression explains, at least in part, the DRG neuron activity representing the activation of the primary afferent neurons (17). The peripheral inflammation with immune response and parasite burden is certainly a contributing mechanism to the activation of these neurons. Spinal cord neuroinflammation also induces a retrograde neuronal sensitization (42). In this sense, we questioned whether the intrathecal treatments that inhibited spinal cord neuroinflammation would also diminish DRG activation because intrathecal delivery of drugs may also reach spinal nerve roots and DRG exerting its effects directly in this site (41). The intrathecal treatments with α-aminoadipate, minocycline, and PDTC inhibited the *T. cruzi* infection-induced mRNA expression of *Cx3cl1*, *Cx3cr1*, *Gfap* (marker of satellite glial cells activation in the DRG), *Tnfα*, and *Il1β* (except by α-aminoadipate). In models, such as paclitaxel-induced neuropathy, infiltrating macrophages produce IL-1β in the DRG site (60), and the glycoprotein 120 of HIV-1 induces enhanced production of IL-1β in satellite glial cells (61). Moreover, minocycline reduced the activation of microglial and satellite glial cells in a model of visceral pain induced by colitis (62). Thus, infiltrating macrophages and satellite glial cells may produce IL-1β in the DRG, depending on disease context and minocycline effect, which seems to be more pronounced in satellite glial cells than α-aminoadipate possibly explaining the significant inhibition of *Il1β* expression by minocycline and not α-aminoadipate. The participation of DRG-neuron derived CX_3_CL1 in the maintenance of nociceptor neuron sensitization during inflammatory pain was previously shown and occurs as a result of a paracrine circuit that involves its interaction with its receptor CX_3_CR1 in satellite glial cells, which, in turn, produces additional TNF-α, IL-1β, and prostanoids that act directly on neurons perpetuating the hyperalgesic state (63). For instance, indomethacin is a COX-1/2 inhibitor (64, 65) that diminished CX_3_CL1-induced pain and PGE_2_ production by satellite glial cells (63). In the present model, *T. cruzi* infection did not increase the mRNA expression of *Cox2* as well as the intrathecal treatment with glial and NFκB inhibitors had no effect on its expression. Thus, suggesting that prostanoids are not major players in the neuronal sensitization in the DRG upon *T. cruzi* infection at the 7th day p.i. It is possible that the different stimulus applied in the models dictates which mediators will be released in greater or lesser quantity and their contribution to the physiopathology of disease.

**Figure 10:**
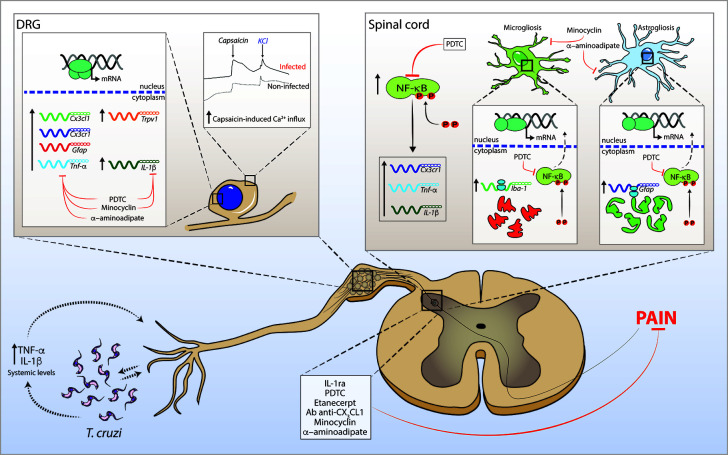
Schematic proposition for *T. cruzi* infection-induced hyperalgesia-related mechanisms in mice. *T. cruzi* infection induces upregulation of systemic levels of TNF-α and IL-1β (and yet undetermined hyperalgesic mediators). The interface between *T. cruzi* parasites and pro-inflammatory cytokines may sensitize nociceptor neurons in peripheral tissue initiating nociceptive neurotransmission. Infection activates DRG cells and promotes increase in mRNA expression of *Cx3cl1*, *Cx3cr1*, *Gfap*, *Tnf-α*, *Il-1β*, and *Trpv1* at this site. In the spinal cord, *T. cruzi* infection leads to the activation of NFκB and increases mRNA expression of *Cx3cr1*, *Tnf-α*, and *Il-1β*. NFκB activation accounts for the gliosis in the spinal cord. These neuroinflammatory events contribute to central sensitization, resulting in increased pain in infected animals. In DRG, treatments with α-aminoadipate, minocycline, and PDTC inhibit the increased mRNA expression of hyperalgesic molecules with the exception of α-aminoadipate that did not inhibit the increase of *Il-1β* mRNA expression. In the spinal cord, α-aminoadipate, minocycline, and PDTC inhibit glial- and NFκB-dependent activities as well as increased mRNA expression of hyperalgesic molecules *Cx3cr1*, *Tnf-α*, and *Il-1β*. Confirming the data of DRG and spinal cord hyperalgesic molecule expression, spinal treatments with Ab anti-CX_3_CL1, etanercept, IL-1ra, α-aminoadipate, minocycline, and PDTC inhibit *T. cruzi*-induced hyperalgesia in infected animals.

In summary, the present data reveals the role of spinal cord astrocytes, microglia, NFκB, and cytokines/chemokines in experimental *T. cruzi* infection-induced pain. Glial reactivity is already detected in the acute phase (7 days) of infection. An early combined contribution of spinal cord astrocytes and microglia that persists until the end of the experimental protocol (28 days p.i.) was observed. To our knowledge, this is the first report unveiling such cellular and molecular pain mechanisms in *T. cruzi* infection. This study opens a novel venue for future studies investigating pain mechanisms and analgesic therapies to improve the quality of life of CD patients. **Figure 10** shows a schematic figure proposing the pathophysiological mechanisms involving *T.cruzi* infection-induced hyperalgesia.

## Data Availability Statement

The datasets generated for this study are available on request to the corresponding author.

## Ethics Statement

The animals were used respecting the protocols evaluated and approved by the CEUA (the Animal Welfare Ethical Review Board) of the State University of Londrina (process number 1067.2015.64). Animal care and handling tasks were performed following the Brazilian Council on Animal Experimentation (CONCEA), the Directive 2010/63/EU for animal experiments, and in agreement with the International Association for Study of Pain (IASP) guidelines.

## Author Contributions

SB designed the study and conducted most experiments, analyzed the data, and wrote the manuscript. VF, TTC, VT, TZ, FP-R, CF, and LS-F designed and performed experiments. RC, WP, FC, TMC, and PP-F contributed with reagents, analytical tools, and expert intellectual support for the study. WV conceived and designed the study, supervised the project, analyzed the data, and wrote the paper. All authors contributed to the article and approved the submitted version.

## Funding

This work was supported by grants to purchase reagents, equipment, and consumable products and bursaries for students from Conselho Nacional de Desenvolvimento Científico e Tecnológico (CNPq), Coordenação de Aperfeiçoamento de Pessoal de Nível Superior (CAPES; finance code 001), FAPESP under grant agreements 2011/19670-0 (Thematic Project) and 2013/08216-2 (Center for Research in Inflammatory Disease), Programa de Apoio a Grupos de Excelência (PRONEX) grant supported by SETI/Fundação Araucária and MCTI/CNPq, and Governo do Estado do Paraná (agreement 014/2017, protocol 46.843), and Programa de Pesquisa Básica e Aplicada da UEL—PBA 2016 grant from Fundação Araucária, Secretaria de Saúde do Estado do Paraná (SESA) and Governo do Estado do Paraná (Brazil). The confocal microscope was acquired by a project supported by Financiadora de Estudo e Projetos (FINEP)—Apoio à Infraestrutura (CT-INFRA 01/2011; process 01.13.0049.00). SB received postdoctoral fellowships from CAPES and CNPq (process 152792/2016-3), and Fundação Nacional de Desenvolvimento do Ensino Superior Particular (FUNADESP; grant number 5301159) research fellowship during nonoverlapping periods of development of this study. RC, WP, FC, TMC, PP-F, and WV acknowledge the CNPq Productivity fellowship.

## Conflict of Interest

The authors declare that the research was conducted in the absence of any commercial or financial relationships that could be construed as a potential conflict of interest.

## Supplementary Material

Supplementary Video 1 3D representation showing that nuclear NFκB is surrounded by membrane GFAP staining.

Supplementary Video 2 3D representation showing that nuclear NFκB is surrounded by membrane Iba-1 staining.
