# Supplementary material for: Experimental Trypanosoma cruzi Infection Induces Pain in Mice Dependent on Early Spinal Cord Glial Cells and NFκB Activation and Cytokine Production
Source: Front Immunol. 2021 Jan 26;11:539086. doi: 10.3389/fimmu.2020.539086 (PMC7870690; doi:10.3389/fimmu.2020.539086)
Supplement: Supplementary file 3 [file DataSheet_1.pdf]

Experimental *T. cruzi* infection induces pain in mice dependent on early spinal cord glial cells and NFκB activation and cytokines production

Sergio M. Borghi,[†,⊥] Victor Fattori,[†] Thacyana T. Carvalho,[†] Vera L. H. Tatakihara,[†] Tiago H. Zaninelli,[†] Felipe A. Pinho-Ribeiro,[†] Camila R. Ferraz,[†] Larissa Staurengo-Ferrari,[†] Rubia Casagrande,[‡] Wander R. Pavanelli,[†] Fernando Q. Cunha,[Ψ] Thiago M. Cunha,[Ψ] Phileno Pinge-Filho,[†] Waldiceu A. Verri Jr[†,]*

[†]Department of Pathology, Center of Biological Science, State University of Londrina, Rodovia Celso Garcia Cid KM480 PR445, CEP 86051-990, Postal Address 10.011, Londrina, Paraná, Brazil.

[⊥]Center for Research in Health Science, University of Northern Paraná - Unopar, Rua Marselha, 591, Jardim Piza, 86.041-140, Londrina, Paraná, Brazil.

[‡]Departament of Pharmaceutical Sciences, Health Sciences Center, University Hospital, Londrina State University, Avenida Robert Koch, 60, 86038-350, Londrina, Paraná, Brazil.

[Ψ]Department of Pharmacology, Ribeirão Preto Medical School, University of São Paulo, Avenida Bandeirantes, 3900, 14049-900, Ribeirão Preto, São Paulo, Brazil.

*Correspondence
Waldiceu A. Verri Jr
waldiceujr@yahoo.com.br; waverri@uel.br

Running title: Spinal glia-related inflammation mediates *T. cruzi*-induced pain.

SUPPLEMTARY FIGURES

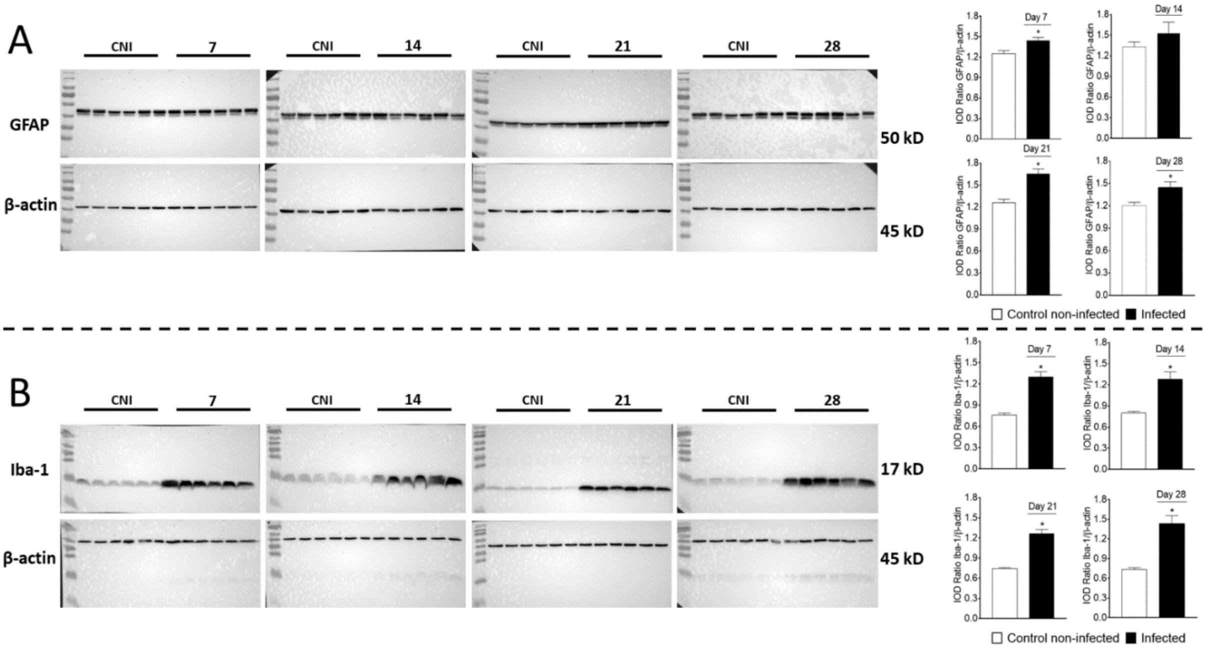

Figure S1. Time-course (7-28 days post-infection) of spinal cord glial cells activation after experimental *T.cruzi* infection through western blot analysis. Panel **A** shows the uncropped original western blot membranes and optic density analysis for GFAP. Panel **B** shows the uncropped original western blot membranes and optic density analysis for Iba-1. * $p < 0.05$ compared to control non-infected mice (*t*-test).

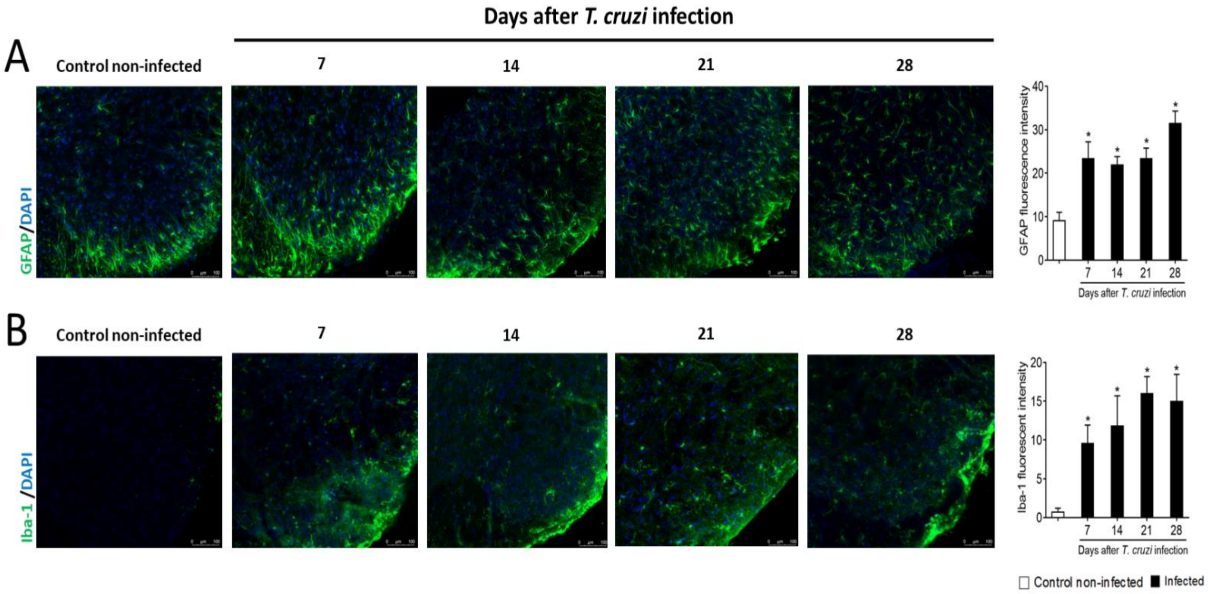

Figure S2. Time-course (7-28 days post-infection) of spinal cord glial activation after experimental *T.cruzi* infection through immunofluorescence analysis. Panel **A** is representative for GFAP and panel **B** is representative for Iba-1. * $p < 0.05$ compared to control non-infected mice (one-way ANOVA followed by Tukey's post-test).
